# Models and Frameworks: A Synergistic Association for Developing Component-Based Applications

**DOI:** 10.1155/2014/687346

**Published:** 2014-08-03

**Authors:** Diego Alonso, Francisco Sánchez-Ledesma, Pedro Sánchez, Juan A. Pastor, Bárbara Álvarez

**Affiliations:** División de Sistemas e Ingeniería Electrónica (DSIE), Universidad Politécnica de Cartagena, Campus Muralla del Mar, 30202 Cartagena, Spain

## Abstract

The use of *frameworks* and *components* has been shown to be effective in improving software productivity and quality. However, the results in terms of reuse and standardization show a dearth of portability either of designs or of component-based implementations. This paper, which is based on the *model driven software development* paradigm, presents an approach that separates the description of component-based applications from their possible implementations for different platforms. This separation is supported by automatic integration of the code obtained from the input models into frameworks implemented using object-oriented technology. Thus, the approach combines the benefits of modeling applications from a higher level of abstraction than objects, with the higher levels of code reuse provided by frameworks. In order to illustrate the benefits of the proposed approach, two representative case studies that use both an existing framework and an ad hoc framework, are described. Finally, our approach is compared with other alternatives in terms of the cost of software development.

## 1. Introduction

*Component-based software engineering* (CBSE), also called component-based development, is a well-known software design paradigm that provides a more flexible way of reuse that overcomes many of the limitations of the object-oriented (OO) approach. CBSE relies on the concept of software component which has been defined in several ways in literature. An early and commonly used definition from Szyperski [1] states that “*A software component is a unit of composition with contractually specified interfaces and explicit context dependencies only. A software component can be deployed independently and is subject to composition by third parties*.” Another common definition by Heineman and Councill [2] states that “*A software component is a software element that conforms to a component model and can be independently deployed and composed without modification according to a composition standard*.”

As established by Lau and Wang [3], the principles of CBSE can be applied in practice either by considering the components as objects or by considering them as architectural units. The benefits of adopting components as objects derive from the broad dissemination of this technology (development environments and tools, available libraries, etc.). However, the OO paradigm defines interaction mechanisms (method invocation) that tend to couple the objects and hinder their reuse. Considering the components as architectural units [4] obviates the above drawbacks, since components are coarse-grained entities with loose dependencies among them. CBSE also facilitates both design for reuse and design by reuse, as it allows the creation of new components by composing previously defined ones. The downside is the lack of development tools and implementation environments, in part due to the fact that the concepts of CBSE can be interpreted in different ways, depending on the context in which they are going to be integrated. This has been a matter of interest for the authors of this paper in the last few years through different research works [5–7].

Despite the expected benefits of using the CBSE approach in software development, there is no complete evidence of its application on a large scale. Although CBSE was originally conceived to be independent of the underlying implementation platform; in the end this has not been the case in practice, due mainly to the two following facts.

- There is no unanimous consensus regarding the concepts, definitions, and rules for the construction of software systems using CBSE [8]. One direct consequence of this is that software component models are not portable between platforms, let alone the software derived from such models. These problems can be clearly seen in the study reported by Lau and Wang in [3], which presents a review of the three most representative component models and concludes that, in general, none of the existing component models are ideally suited to fulfill the promises made by CBSE.
- Each of these component models is linked to a specific implementation, so that choosing one implies a choice of implementation. This is not bad in itself, provided that the implementation exhibits the characteristics expected from the component-based design. However, the implementation includes characteristics that do not appear explicitly in the component model (e.g., concurrency management) and that are generally unfamiliar to CBSE application developers who may lose control over some characteristics of the application.

Consequently, the development of applications based on a purely CBSE approach, where components are considered architectural units, still requires solutions to the problem of defining and implementing a series of mechanisms to translate CBSE designs into executable programs (i.e., a compiler for a component-based language). The authors of this paper maintain that the* model driven software development* (MDSD) paradigm [9, 10] can provide the theoretical bases and the tools necessary to solve such challenge. Currently, MDSD is one of the mainstream paradigms in software engineering. MDSD is a synonym of the OMG model-driven architecture [11] initiative. However, in the formal methods community, MDSD is seen as model-based techniques for software design and verification. Because of the difference between the nature of research and practical model-driven software engineering, there is a gap between formal techniques, together with their tools, and their potential support to practical software development. In order to bridge this gap, [12] defines meanings of component-based software architectures and shows how can be modeled using a formal MDSD method. Although relatively novel, MDSD has produced promising results in applicable domains such as automotive, aviation, and electronics industries, among others [13, 14]. These results have been quantified in terms of improvements in levels of reuse, quality of the resulting software, and reduction of lead times in development.

Additionally, in terms of software implementation,* frameworks* [15] offer the highest level of code reuse and flexibility, given that it is possible to consider them as semicomplete applications which are specialized to produce concrete applications. A framework embodies the features common to many applications in the domain of interest and at the same time offers specialization mechanisms and points of variability with which we specify differences.

This paper describes a generic approach that combines the use of the abovementioned resources, that is, components, models, and frameworks, to develop software applications that overcome the previously described deficiencies. We demonstrate the proposal through the development of two case studies: the integration of an existing general-purpose framework as well as the development and integration of a new framework for real-time applications. On that basis the paper is structured as follows.  Section 2 gives an overview on research and the most significant related work that has been done in the field.  Section 3 details the proposed development approach for CBSE applications, which is illustrated in Section 4 with the aforementioned case studies.  Section 5 is devoted to discuss the approach in terms of development effort, and finally Section 6 presents the conclusions a future works.

## 2. An Overview of Research

The purpose of this section is to review what is already known about the research topic as a whole, and to outline the key ideas and theories that help to understand the contribution of the work. For that, some of the available research works related to the software engineering disciplines used in this paper are described and briefly compared.

### 2.1. On Models and Components

Numerous authors have concluded that a high degree of reuse and standardization is necessary to keep increasing quality and develop systems in a cost-effective manner. One possible solution is to design a process aimed at producing high quality, reusable, verified, and validated components. The works cited here are representative of the existing related works in the literature and they are detailed next with the aim of illustrating the interest of researchers in adopting MDSD as the basic approach to develop component-based software systems.

As stated by [16], MDSD is a promising approach to address platform complexity given the inability of third generation programming languages to alleviate this complexity and express domain concepts. To tackle this challenge, MDSD considers* models* as first-class artifacts [17]. Thus, a system can be represented at a certain level of abstraction using models as simplified representations of reality in which nonrelevant details are abstracted, thus improving both comprehension and communication of the reality underlying these models. With the right selection of levels of abstraction it is possible to separate software artifacts that are independent of the implementation platform from those that are not. Models are therefore the main artifacts driving the development process. Models are defined according to* metamodels*, which embody the concepts relevant to a particular domain of application, and also the relationships between them. In other words, metamodels represent the abstract syntax of the language. Model transformations [18, 19] are also key mechanisms in MDSD, since they allow developers to define the way in which these models will be interpreted and transferred to other representations (with a different metamodel) at the same or a different level of abstraction, until code is generated in a programming language.

MDSD has been successfully applied with different purposes and in different domains as fault-tolerant systems [20], user interfaces [21], multiagent systems [22], game development [23], and systems engineering [24], among others. In all of them, MDSD appears as a promising software development approach that aims to change the focus of software development from code to models by enabling systems engineers to model their domain knowledge and tooling on a more abstract level.

Components are normally used as the building blocks of the application architecture, since the abstractions they provide are better suited for this purpose. However, the terminology can be sometimes confusing, and thus it is worth clarifying it. According to the Software Engineering Institute [25], a* component model* is the set of component types, their interfaces, and, additionally, a specification of the allowable patterns of interaction among component types. This differs from what is therein called* component framework*, which is the infrastructure that supports the component model on the target platform.

A classification framework proposing a taxonomy and terminology for studying component models is presented in [8]. Its main conclusions are that (i) the vast majority of the reviewed component models take into account platform details, (ii) deployment configuration of the application is mainly done at compile time; thus, no reconfiguration is possible at run time, (iii) nearly almost all of the component models consider required and provided interfaces, (iv) almost all of them differentiate between interfaces for data exchange and for control flow, (v) most of these models only deal with the structural modeling of the application, leaving the implementation of the logic of the components (and hence their behavior) to the manual codification stage, (vi) in most of the component models, the dominant interactions styles are request/response and “pipes & filters,” while the most adopted communication type is synchronous communication, and (vii) nearly half of them manage extrafunctional properties like timing properties and resource usage (CPU, memory, I/O, etc.).

Considering such characteristics and given the number of reviews of the state of the art of CBSE in the literature, our aim is not to provide a new review, but to relate the work described in this paper with proposals where CBSE is the approach and MDSD is part of the solution.

Many authors have recognized the advantages of combining models and components. A component-based development process using model-based development principles aimed at producing reusable components for embedded on-board space applications is described in [26]. On other domains, as, for example, robotics, developers have created component-based software frameworks to support the development and reuse of pieces. For instance, the BRICS component model [27] provides robotics developers with a set of tools for developing components and component-based architectures using some existing frameworks in a MDSD context. Reference [28] applies component-based technologies to MDSD for improving the reusability of models and automatic generation efficiency. In the proposed approach, relevant model elements are packed as a component, and modeling software is carried out associating with the model components. A MDSD approach to progress on the successful route to service-oriented architecture (SOA) engineering with minimal design decisions losses is described in [29]. The authors propose a model-to-model transformation that preserves the architectural alignment between the business process and their supporting service implementation infrastructure. The method relies on development scenarios where long-running business service composition models drive their supporting service implementation models. The introduced mapping rules and their implementation in a proof-of-concept prototype enable the automatic transformation of business models to service component architecture assembly models. Thus, it promotes the separation of business concerns, enabling quick evolutions of the IT infrastructure.

CBSE and MDSD techniques are also being used to design repositories where services are located to configure components at runtime. An approach for the generation of platform specific models, inspired by the selection processes of COTS components and being applied in the domain of component-based graphical user interfaces, is described in [30]. Rouvoy and Merle [31] combine model and component paradigms to reach more adaptability in the implementation of middleware. In particular, they present a framework that uses models to describe middleware standards and compile them to components. In [32] a component-based model-driven architecture that gives full flexibility of the automation in source code generation for power system software applications development is presented. The main idea of this work is to connect expert knowledge in power systems with the ongoing software development initiatives and make it later available to power system experts that usually lack software development knowledge. A combination of CBSE and MDSD where components are assembled to quickly build the software system is proposed in [33]. In particular, the approach includes the implementation of software systems through the design and realization of components assembled from platform-independent models.

Many component technologies exist today to solve deployment and reconfiguration issues offering ad hoc solutions. DYVA [34] is a unified framework for dynamic reconfiguration of component applications in order to help in developing specific hot deployment and reconfiguration systems by the personalization of such unified framework. The framework is targeted to existing component models and allows developers to focus only on the business logic of their systems. The approach has been validated for JavaBeans and OSGi [35] models.

On the industrial side, over the last years the Software Group of the Motorola Networks and Enterprise Division has engaged in establishing an MDSD approach to allow the efficient creation of quality software to be used in a variety of product lines [36]. This work concludes that, undertaking an approach to large scale, component based model-driven software development is clearly a major endeavor, of which the organization has been benefited greatly and has improved the productivity and efficiency in the development of its products.

### 2.2. On Frameworks

Frameworks are software artifacts specifically designed to promote reuse. More general and innovative proposals have recently appeared in the literature, focusing on the use of frameworks for software systems development.

Fairbanks et al. [37] propose a method for specialization of OO frameworks using design patterns, which provides a design fragment for the system as a whole. A design fragment is a proven solution to the way the program should interact with the framework in order to perform a function. The idea is for each framework to have its own catalog of design fragments, offering conventional solutions to known problems. The proposal is validated with an eclipse-based tool containing more than fifty patterns. Antkiewicz et al. [38] go a step further by providing a conceptual and methodological framework for the definition and use of framework-specific modeling languages (FSMLs). An FSML is an explicit representation of the specific features of a domain as offered by the associated framework. Thus, FSMLs can be used to express framework-specific application models. These models describe the instances of the features supplied by the framework that are ultimately implemented in the application's code.

Some authors as Cervantes et al. [39] defend that frameworks must be considered as first-class design concepts during architectural design. They describe an approach to architecture design where frameworks directly appear in the steps of the method attribute-driven design (ADD) used to perform application design. That is, they state that frameworks should not be relegated to the last phases of the development process but rather be considered from the very beginning.

These works very clearly illustrate the trends in framework development and should therefore be borne in mind in any proposal in that connection. In short, frameworks based on OO platforms and languages have limited scalability and extendability owing to the restrictions imposed by the use of objects [40]. Furthermore, component-based frameworks improve scalability and extendability by providing mechanisms to facilitate dynamic reconfiguration of components, even at execution time. Thus, component-based frameworks facilitate integration and interoperability if they are built in such a way as to make allowance for future extensions by the addition of new components.

As a summary of this section, the research presented in this paper contributes to the existing state of the art by providing a novel synergistic combination of models, components, and frameworks where we have the following.

- *The modeling of the applications is strictly CBSE*. The modeler does not need to know any details of the implementation of the component model since these details will be part of the selected framework that will eventually support the platform.
- *The proposal is supported by MDSD technology*. The use of models, as well as increasing the level of abstraction, makes it possible to integrate other models to carry out early validation and verification activities and to postpone the choice of platform characteristics given the possibilities of automatic code generation.
- *The implementation is supported by the reuse of frameworks*. The developer perceives each framework from a purely conceptual standpoint through the set of characteristics that define it. These characteristics can be instantiated as part of the framework specialization process. Thus, the choice of framework will depend on the set of characteristics it offers (e.g., distributed components as opposed to a centralized schematic, communication layouts, etc.). The selection of the target platform is postponed until as late as possible.
- *Complexity is hidden to developers*. The application developer views each framework as a set of “hot-spots” that can be filled in from the application's architectural component model. The automated model-to-model transformations of MDSD conceal the complexity of the framework from the developer. This is a considerable advantage since the learning curve associated with frameworks is generally long.

## 3. Description of the Approach

As said before, the approach relies on the use of models and frameworks for developing component-based applications. The main ideas are that applications are modeled by means of a component-based modeling language, following MDSD principles, while the concrete platform details are provided by a component-based software framework. In this work, we use specifically the component diagram semantics of UML 2 [41], though any modeling language could be used to describe applications, as long as it provides the primitives required to model component-based applications.

At the same time, frameworks are semicomplete application skeletons that offer (i) a common software infrastructure and run-time support for a range of applications that share some requirements and (ii) concrete software extension points (normally entitled “hot-spots,” that use mechanisms like class inheritance, object composition or subscription, etc.) where the user has to add the application-specific code. Frameworks can be designed to ease the creation of any kind of software application, such as graphical interfaces (well-known examples are Java Swing or Qt (Qt homepage: http://qt-project.org/) frameworks), communication middleware (like ICE (ICE homepage: http://www.zeroc.com/ice.html) or ACE (ACE homepage: http://www.dre.vanderbilt.edu/~schmidt/ACE.html)), and so forth. Particularly, we are interested in the use of* component frameworks*, that is, frameworks that support the concept of component as a unit of architectural design and composition, independently of the way they are finally implemented (classes, functions, etc.). Examples of component frameworks are the Open Services Gateway Initiative (OSGi) [35] and Enterprise Java Beans (EJB) (EJB homepage: http://www.oracle.com/technetwork/java/index-jsp-140203.html), to mention but just a few.

A set of model transformations complete the approach by generating the code required to instantiate the target framework's “hot-spots” from the input model. For instance, if the framework provides a base class for implementing components (class “Component”), the transformation will generate a class skeleton that inherits from “Component” for each component in the input model. The user has to complete the generated classes with the code that implements the component specific functionality afterwards.

In the proposal, the application code is organized and partitioned into three groups, namely, (C1) code supplied by the implementation support in accordance with domain requirements, (C2) code that provides an OO implementation of the CBSE concepts and the framework “hot-spots,” and (C3) code that provides the application-specific code. These code groups were defined by us because they help us provide advice on how to develop a new framework and reason about the proposal. But they are generally developed by members of the development team that play different roles and are not always identifiable in each and every use of the proposal. Before fully describing the proposal, we will outline some previous background that justifies it.

### 3.1. Previous Work and Motivation for Adopting a New Approach

Once the approach has been briefly introduced, the first question is why not generate all the code; that is, why do we instead target a framework. That is the normal approach when using MDSD techniques, where code generation is normally postponed until the final stages of the process, when a model-to-code transformation is carried out on a refined model containing the bulk of the details of the implementation. This was the approach that we followed in [5, 42], from which we extracted some conclusions that pointed clearly to the need for research into new solutions that promote reuse and ease maintenance. In those articles, we applied a model transformation to generate nearly the 70% of the Ada code required to control a Cartesian robot, whose architecture comprised ten components. The transformation generated a total of sixty-five Ada classes which together implemented around six design patterns from [43], needed to implement software structures that behave like components. Though the number of generated classes for such small example may seem a bit high, it is not particularly relevant, similarly as nobody is in general concerned about the assembly code generated by a C++ compiler, as long as its behavior is equivalent to the original C++ code.

What is really important and a limiting fact of the fully generative approach is the nature of model transformations. They do not usually have a modular structure that helps create and maintain large transformations, which also prevents them from being reused (totally or partially) in systems that may have similar requirements. Also, transformation rules can exhibit a high coupling degree among them, where a given output element is partially generated from different rules. Thus, a transformation that aims at generating all the implementation code must generate both the complete infrastructure that implements the support needed to execute entities that behave like components, as well as the concrete application code. It is thus a rather complex software artifact that is not easy to understand, maintain, or develop further.

**Figure 1:**
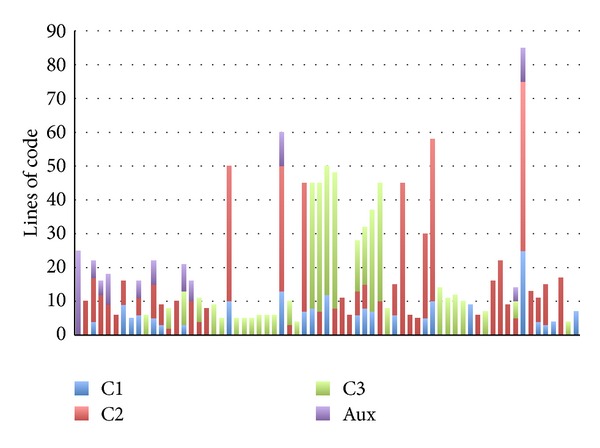
Summary of the lines of code of the model transformation developed for a full generative MDSD approach. Bars correspond to rules, while colors represent the group the rule generates code for.

**Figure 2:**
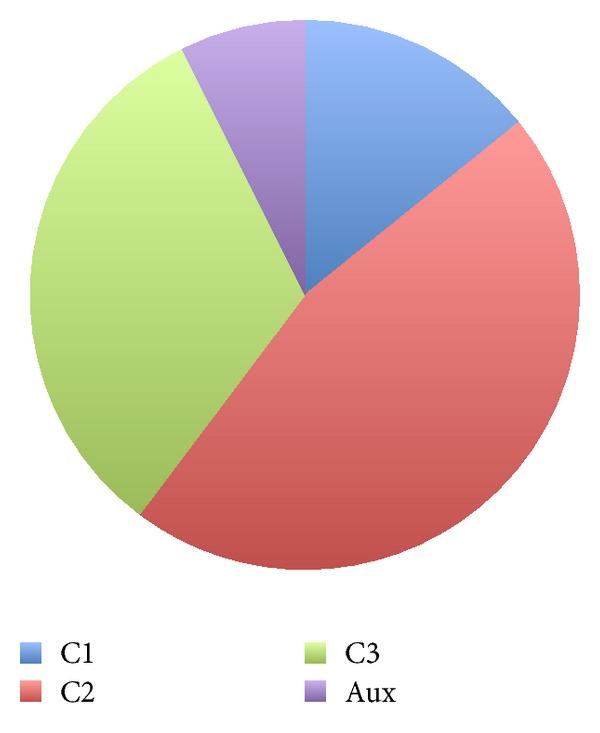
Pie chart showing the distribution percentage of the lines of code involved in the generation of each kind of code group.

Figures 1 and 2 support the aforementioned conclusions by summarizing the size and complexity of the model transformation we developed in [5]. Each bar in Figure 1 corresponds to a rule of the transformation, while its color represents the number of lines of code that are devoted to create the classes that provide the infrastructure runtime (C1), implement CBSE concepts (C2), generate application-specific code (C3), and generate elements needed by the transformation itself (Aux), respectively. The first and second bars, starting from the left, group together many small rules (only one or two lines in length), while the remaining sixty-five bars represent one rule each. The order of the rules is not important since model transformation languages are declarative. It can be noted that thirty-eight rules generate code for just one of the considered code groups (C1, C2, or C3), twenty-four rules target two groups, while only two rules generate code for the three of them. That is, the transformation exhibits a medium coupling degree between rules, since less than half of the rules generate code for more than one group.

Figure 2 shows the distribution of lines of transformation code devoted to generate each kind of code (C1, C2, and C3). As can be seen, nearly two thirds of the total number of lines are related to the generation of the runtime infrastructure and the implementation of CBSE concepts, which are independent of the concrete application being generated (which is in fact represented by C3). This led us to conclude that we could simplify model transformations by leaving only the rules needed to generate C3, while using component frameworks as the target platform that embeds C1 and C2. This decision also makes it possible to reuse already existing and tested frameworks, since in this case only a transformation that generates the code needed to instantiate the framework “hot-spots” is required.

### 3.2. The Proposal

**Figure 3:**
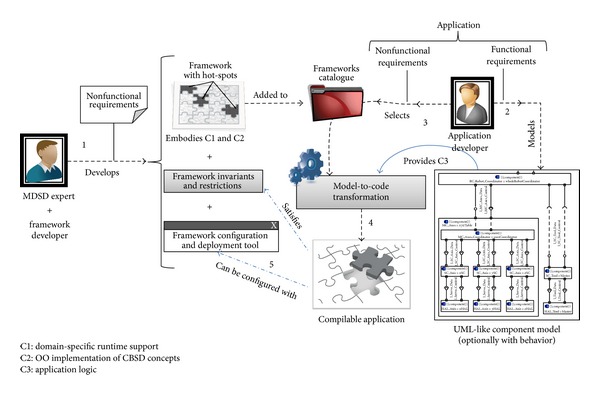
Proposed development process for CBSE applications, based on MDSD technologies for designing and validating applications, and on frameworks for providing the required run-time support. Three developer roles are shown in the figure: application developer, MDSD expert, and framework developer. The application code is organized into three sets: code providing the runtime support (C1), code providing the implementation of CBSE concepts in an OO language (C2), and code providing the application-specific functionality (C3).

Figure 3 shows a schematic view of the proposal described in this paper, which encompasses and integrates CBSE and MDSD approaches in a development context based on the use of frameworks for the reasons described in the previous section. The approach distinguishes three types of role:* application developer*,* MDSD expert*, and that of* framework developer*.

The application developer designs a concrete component-based application starting from its functional requirements (step 2 in Figure 3) and using an architectural component-oriented modeling language, like UML 2 [41] or similar ones. Once the application has been modeled, the developer selects the framework (step 3 in the Figure) that supplies the nonfunctional requirements, instantiating the framework's “hot-spots” through a model-to-code transformation (step 4 in the Figure), and optionally using the application configuration infrastructure in case the chosen framework provides one (step 5 in the Figure). This configuration infrastructure, when available, provides a fine-tuning capability to set aspects that are not possible or not desirable to define at an architectural level, like setting the number of threads or their timing characteristics, communication policies, network parameters, configuration file paths, and so forth.

The idea is that code C3, that is, code providing the application specific functionality, is generated and integrated automatically by means of model transformations, generated from models that are independent of the implementation platform, and this prompts our claim that “*models fill hot-spots in frameworks.*” In other words, the selected framework details are totally hidden for the application developer, who views it from a conceptual standpoint, independent of the platform, as a set of “hot-spots” that will be filled in from the application's architectural component model by means of a model transformation.

It is worth noting that the model built by the application developer should take into consideration any nonfunctional requirements that can be explicitly represented in the architecture. For example, reliability can be achieved by means of redundant components, which should be explicitly included in the model. At the same time, the framework must support all requirements that can be expressed in the architectural model plus any that cannot be explicitly represented in it. For example, supposing there should be real-time requirements, even though implementation threads are not defined at an architectural level, it is assumed that the threads supplied by the framework can be analyzed to verify the schedulability of the OO application generated. The developer will be guided by this and other characteristics when selecting one framework over others.

In case none of the available frameworks provides the application nonfunctional requirements, the approach considers that a new framework should be integrated, either by creating a model transformation that targets an existing framework or by developing a new framework and the model transformation required to use it. In both scenarios, it is the role of the* MDSD expert* to develop a model transformation that generates the application-specific code (code C3) by using the “hot-spots” provided by the new framework. In this case, code belonging to groups C1 and C2 (code providing the runtime support and the implementation of CBSE concepts) is not normally isolated or identifiable, but rather it is mixed up in the framework code. If the framework has been properly designed for supporting component-based applications, it will surely provide the “hot-spots” required to correctly implement the applications.

In case a new framework should be developed, starting from a domain engineering approach, the* framework developer* extracts and organizes a set of specific common characteristics of applications in the domain of interest. On the basis of these characteristics and nonfunctional requirements, we propose that the developer should clearly separate the code of the framework he/she is going to develop (step 1 in Figure 3) into the following groups: (i) the implementation infrastructure to support these applications (code C1), (ii) an interpretation of the CBSE features in OO technology (since this is the chosen implementation technology, code C2), and (iii) a set of “hot-spots” to support application variability and evolution, embedded in both C1 and C2. Code C2, as well as supplying the OO interpretation of the CBSE features, reduces the existing coupling between C1 and C3, thus allowing them to evolve separately. In this way it will be possible to reuse the same C1 implementation support in different applications as long as C2 is kept constant. At the same time, this facilitates reuse of the same application (component model) with other implementation requirements (same C3, different C1). This flexibility is achieved at the cost of introducing some coupling between C1 and C2.

As shown in Figure 3, the framework developer has also to define the restrictions the framework imposes on the final application, like forbidden combinations of classes or structures, bad practices, forbidden language constructs, and so forth. He/she may optionally provide the framework with a configuration infrastructure enabling the user himself/herself to modify or tune in the final application, within certain limits.

## 4. Demonstration of the Approach with Two Case Studies

In order to show the feasibility and flexibility of the proposed approach, this section illustrates its use with two representative case studies that use both an existing framework (the iPOJO implementation of OSGi [35]) and an ad hoc framework developed by us. The same example is used in both case studies. For simplicity and concreteness it only comprises two components with a client-server relationship, where the functionality invoked by the client does not change, but the nonfunctional requirements change in a way that led to the selection of a different framework.

The service exposed by the server consists of a collision detection algorithm that receives starting and ending points and calculates the distance to the closest obstacle in the trajectory from the start to the end points. It is not difficult to generalize the example to include more interfaces, services, and components. The examples are also aimed at the identification of C1 (runtime-support), C2 (object-oriented interpretation of CBSE concepts), and C3 (user specific application code).

### 4.1. First Case Study: Simple Client-Server Application

**Figure 4:**
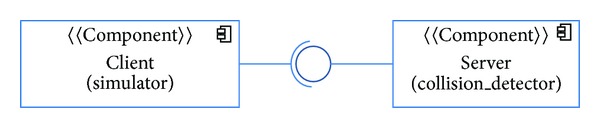
The sample application in terms of components, connections, and interfaces (structural view).

Consider a simulator application that needs to use the aforementioned collision detection service. In this case, (i) the simulator can be blocked and patiently wait for the server to response, (ii) the simulator only has one operational mode, and (iii) there may be multiple servers running concurrently (the number of server components can be increased if the number of simulators increases), but there is no concurrency on servers. For simplicity we assume that the example comprises two components that are connected by means of an interface that defines the aforementioned service (see Figure 4). We could generalize the example to include more interfaces, services, and components, providing that the above list of requirements remains unchanged.

As we will see, an application of this nature can be easily developed through the iPOJO framework that implements the OSGi component model.

#### 4.1.1. The iPOJO-OSGi Framework

OSGi is a specification of a component model suited for any project interested in the principles of modularity, component orientation, and/or service orientation. It defines a dynamic service deployment framework that is amenable to remote management and facilitate the “componentization” of Java software modules and applications.

Among the available implementations of this standard we selected Apache Felix (Felix homepage: http://felix.apache.org/) from the Apache Foundation. Felix aims at developing and implementing the OSGi R4 Service Platform and other interesting OSGi-related technologies. Felix comprises many subprojects, but we focus on iPOJO, which provides the core implementation of a service-oriented component model that simplifies OSGi-based development. iPOJO is based on the concept of POJO (*Plain-Old Java Object*), to develop the application logic, while nonfunctional properties are just injected in the component at runtime. iPOJO is an extensible framework, which manages both component bundles and registered services, as well as component's lifecycle. It uses Java bytecode injection to fill in dependencies at runtime, while at the same time it also permits the dynamic creation of components and services. Felix embeds and provides us with the code we called C1 and C2, though both sets are entangled and cannot be isolated. But, what is important, Felix provides the required “hot-spots” to create components and configure itself when needed.

As happens with the vast majority of available commercial component models, iPOJO is a purely structural component model. It provides the runtime support for managing services and components, for connecting required services with provided ones, and for controlling component's lifecycle, but it is not concerned about component behavior nor does it impose a given one. It leaves these decisions to component developers. Thus, in case the input component-based modeling language supports behavior modeling (in the form of state machine, activity, or sequence diagrams), equivalent Java code should be manually added by the developers after the transformation is executed.

#### 4.1.2. Application Development with iPOJO

A programmer that directly uses iPOJO to implement the application would have to manually develop the Java code shown in Algorithms 1, 2, 3, 4, and 5, where (i) CBSE interfaces are implemented as Java interfaces (in this case, the I_Collision interface), (ii) data types are implemented as classes (class Point), (iii) components are also implemented as classes (classes C_Server and C_Simulator), (iv) required interfaces are part of the interfaces implemented by a component, (v) provided interfaces are implemented as private attributes of the appropriate interface type, (vi) active components provide a method to be executed (method run() in class C_Simulator), and (vii) an XML file defines the information Felix needs at runtime to perform bytecode injection (set the attribute portCollision to an instance of class C_Server), as well as to execute at least one of the components.

We have developed a model-to-code transformation that generalizes the aforementioned process in order to integrate iPOJO in our proposal. The transformation generates the following artifacts in the eclipse environment.

- A Java project that contains the definition of all the applications services (both provided and required) as well as their data types. The application developers have to complete the details afterwards. This project also contains an Ant file and a configuration file for Java BND, which together manage the creation of the OSGi bundle.
- A Java project with dependencies on the aforementioned project is created for each component. This project contains a Java class with the component skeleton, where the application developers add the Java code that implements the component behavior. Additional classes can be created by the developer if needed. The project also contains an Ant file, a configuration file for Java BND, and a iPOJO task for Ant configuration file. These files manage the creation of the OSGi bundle and define the points where Felix will inject the dependencies at runtime.

After completing the components' behavior, the developer can generate the OSGi bundles by running the Ant tasks, which will generate a jar file that contains the bundle for Felix. These bundles can afterwards be loaded in the Felix runtime in the correct order: first the bundles that provide the implementation of the data types and services and then the bundles that contain the components.

The restrictions imposed by the Felix implementation of the OSGi platform are the following. The first is that a component cannot offer the same interface in more than one port, since it is not possible to repeat an interface in the  implements part of a class definition. Thus, it is not possible to control which component makes the request, or to have different implementations of the same service, since all service requests arrive to the component through the same point. But the most important and restrictive limitation, from our point of view, is that Felix provides no control over port connection. This connection is performed by the runtime as soon as the provided service a required one is waiting for is added to the system; this is done without even asking the user for confirmation.

Resuming the roles defined for the proposal shown in Figure 3, from the* application developer* point of view, he/she has to (i) define the component diagram of the application, comprising both component, interface, and datatype definitions, (ii) run the transformation, (iii) implement the methods defined in the interfaces as described, and (iv) execute the Ant tasks in order to generate the OSGi bundles. From the perspective of the* MDSD expert*, in this case he/she has to only develop a rather simple model transformation. Given the design of iPOJO, the correspondences between CBSE and OO concepts are nearly direct, one-to-one, which led us to the design of a linear transformation, where the rules are slightly connected and have little dependences among them. The developed model transformation comprises around ten rules and eighty lines of code. In this case, the* framework developer* role is not needed.

We can conclude that, in the same way as iPOJO hides the details of the component implementation, the model transformation hides the details of the iPOJO implementation. Therefore, our proposal provides an additional abstraction level that makes the development process easier.

### 4.2. Second Case Study: An Application with Concurrency Requirements

Consider now that the client of the collision detection service is a controller component. The server functionality is the same, but the client (i) has to accomplish various tasks simultaneously, (ii) exhibits a reactive behavior that depends on the environment, (iii) has different operational modes with different behaviors, and (iv) cannot be blocked by the server nor assume it is properly functioning. These requirements are typical of control applications with real-time constraints. These applications have to be adapted to different types of restrictions (time, energy, interaction with their environment, etc.) and be integrated with other programs, communications infrastructures (e.g., middleware), and so forth.

To be more concrete, the operation of the new application could be as follows. The controller moves a robot in a given environment, following a trajectory, while the server has a description of its kinematics and a map of the environment. The controller periodically sends a query to the server with its current position and the estimation for the next period, so that the controller can generate a new trajectory or stop the robot in case the server detects a potential collision. For safety reasons, the controller has two operational modes: autonomous and manual. The autonomous mode relies on the proper functioning of the server, while the manual relies on a human operator to control the robot. The manual mode is activated when the controller does not receive an answer from the server or when the answer is out of range.

**Figure 5:**
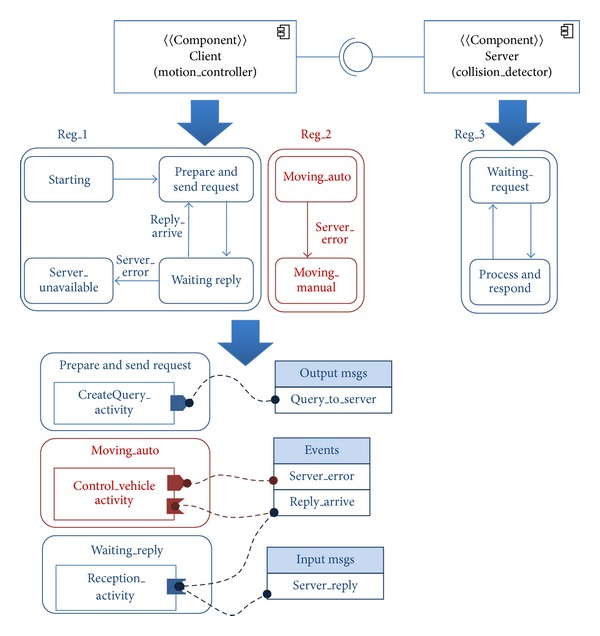
Architecture modeling of the new version of the application in terms of the components' structure and behavior.

Reactive behavior, operational states, concurrency, and timing properties are very important issues that have a great impact on the architecture of the application code, and therefore they have to be explicitly modeled to guarantee the correctness of the application. Thus, we considered additional UML 2 diagrams to cope with reactive behavior and algorithm specification, together with the elements needed to coherently relate them. Besides the component diagram for expressing component structure, we selected state machines to model component reactive behavior and express potential concurrency by means of orthogonal regions, actions, activities, and pins from the activity diagram to model algorithms as black boxes. The three diagrams are linked together by means of messages: port input messages can generate events for the state machine or contain information for the activities, while activity output messages can generate events for the state machine, be sent to other components through a port, or be sent internally to other activities. Considering the new application requirements and modeling elements, the updated version of the application architecture is shown in Figure 5. The iPOJO model only considers the structural part of the application (top of the figure).

Under these new requirements, the use of iPOJO is much less attractive than in the previous case, although it is still better than starting from scratch. The application developer should address the complex work of programming concurrent tasks and state machines and integrate them coherently. A transformation that generates such code would free the developer of this work but, as shown in Section 3.1, it is far from trivial. Another solution is to extend iPOJO with new classes or interfaces that support the new requirements. The modeling of concurrency in Java is greatly facilitated by the package  java.util.concurrent, but the challenging issues of coding the state machines and their integration with concurrency issues still remain. For these reasons, we decided to develop an ad hoc framework called FraCC.

FraCC has been implemented in the C++ language and has been validated in the implementation of Teleo-Reactive programs [6], as well as in the development of the control software of an underwater vehicle [44]. A preliminary version of FraCC is described in [45].

#### 4.2.1. The FraCC Framework

The fundamental characteristic of FraCC is that it provides an execution infrastructure that supports all the modeling primitives described above. Additionally, it provides control over concurrency policy (e.g., one thread per activity, a single thread for all activities, or any other distribution) and control over the policy of distribution of components in computational nodes. As FraCC has been designed to put into practice the approach described in this paper, the code corresponding to runtime support (C1) and the code that translates CBSE concepts to OO constructions (C2) are clearly identifiable. The application specific code (C3) is in part generated by a model transformation and in part provided by the application developer.

FraCC includes two model transformations that have the application model as input and generate the C++ code skeletons of the algorithms embedded in activities and a description of how FraCC will execute the components (deployment model), respectively. FraCC also includes a runtime module that parses the deployment model, instantiates the application, and links with the user code, in an analogous manner as is done in Felix. More specifically, consider the following.

- The first model transformation generates the C++* code skeletons* where the user should add the application-specific code. The code is then compiled into a dynamic library and loaded by the FraCC runtime. FraCC imposes some restrictions on the code contained in an activity; for example, infinite loops are forbidden, and activities cannot spawn new threads or create mutexes. Therefore, the approach provides a clear separation of concerns, differentiating component modeling from activities implementation.
- The second model transformation generates a* deployment model*, which describes how the application will be executed. This model enables users to distribute the application in threads, processes, and nodes, by assigning components to processes and their algorithms to threads. Note here that FraCC does not give any guidance as to the number of threads that have to be created or how activities should be assigned to threads, but it provides the necessary mechanisms to enable the user to choose the appropriate heuristic methods, for example, the ones defined in [46]. The FraCC runtime uses this model as input in order to instantiate the application. Therefore, FraCC provides a clear separation between architecture and execution, allowing users to define and test different execution and concurrency schemes for the same application architecture.

#### 4.2.2. Design of the FraCC Framework

**Figure 6:**
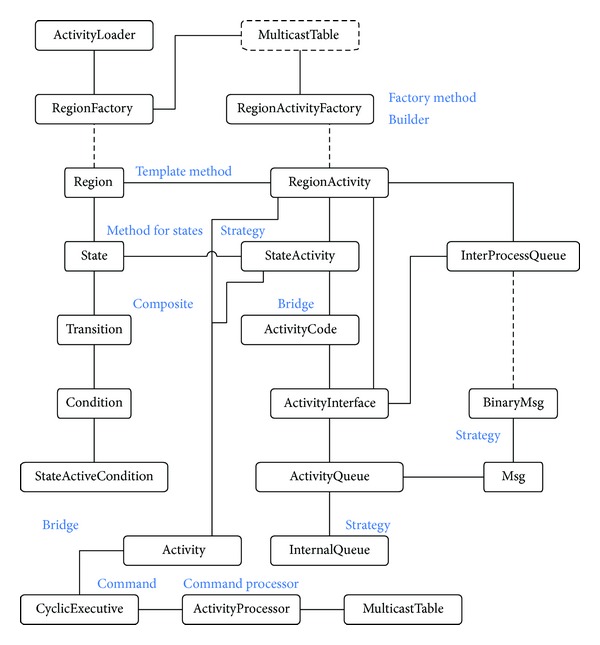
Class diagram of the FraCC framework with some of the design patterns employed in its development.

The design and implementation of a framework are far from simple, but it can be justified by the number of applications that can be developed with it. Nevertheless, framework design, in general, is affordable since there is a wide knowledge body about framework design in the literature in the form of design patterns. Also, new frameworks can be implemented at a great extent by using other existing frameworks. For illustration purposes, a simplified class diagram of the FraCC design is shown in Figure 6, which also includes the main design patterns employed. All the design patterns mentioned in this section are fully described in [43, 47]. The remaining of the section is devoted to describe the main features of FraCC design and to classify them in C1 or C2.

*From Activities to Threads: Concurrency Design (C2).* The main requirement guiding the framework design is that it must provide the user with control over application concurrency, in terms of both the number of threads and their temporal characteristics, so that compliance with them can be verified. For this reason, the framework design started and was conditioned by the way in which concurrency should be managed. FraCC uses the orthogonal regions of the input models as the unit of workload for threads. Threading design relies on the  Command and  Command Processor patterns, where a command processor is a thread that has a list of commands to be executed according to some policy. In FraCC, regions are implemented by means of two classes: one that stores the structure of the region and one that stores its behavior and the behavior of the contained states. The latter plays the role of commands. Other patterns are used to fine-tune the design, such as the  Template Method to organize the management of regions and  Strategy to define their execution policy.

*From Activities to Threads: Support for Concurrency (C1). *C1 comprises the libraries and code to implement threads, shared buffers, and synchronization policies. Buffers store messages exchange among components, as well as events waiting to be processed. Currently, the implementation relies on POSIX primitives, but other alternatives are possible. For instance, we are now considering migrating to the new C++ 11 standard, since it now includes built-in threading support. It is also possible to use higher-level solutions like the ACE framework.

*From Ports to Buffers: Message Delivery Design (C2).* Ports are only used at model level to check that components are properly connected, but they have no direct representation in FraCC. Instead, ports are transformed into proxies of the buffers of the component that receives the message. These proxies hide the actual location of the destination buffer, since components can be distributed in different nodes. Activities use these proxies to send messages to component connected to that port.

*From Ports to Buffers: Support for Message Delivery (C1). *FraCC relies on POSIX shared memory mechanisms when messages are sent between components residing in the same node, and plain sockets otherwise. The implementation uses the  Bridge pattern to support future changes in the communication software (like integrating CAN bus, for instance). As in the previous case, it is also possible to use higher-level solutions based on well-known middleware like ACE or ICE.

*From State Machines to Maps: State-Machines Design (C2). *Like ports, state machines as a whole do not have a direct representation in FraCC, but instead only their individual regions do have one, in the form of commands for command processors. Regions process events, change state if necessary, and then execute the activity associated to its active state, if any. This management process is organized by means of a  Template Method that defines the sequence of actions that implement the execution semantics. The implementation semantics followed is the one defined by UML 2 “run to completion.” If it is needed to change that semantics, all that is required is to change the class implementation. The concrete code of the activities is modeled according to the  State pattern.

*From State Machines to Maps: Support for State-Machines (C2). *FraCC relies on POSIX mechanisms to prevent data corruption in the data collections (mainly hash maps). We have added support for safe data sharing in the C++ STL collections. As can be noticed, the support for data collections and safe threading access appears in many of the above concerns, since messages bound the structural and behavioral parts of a component implementation.

#### 4.2.3. Application Development with FraCC

For comparison purposes, we will now describe the development of an application using FraCC so that we can highlight the similarities and differences with the iPOJO case. From the developer point of view, he/she has to (i) define the component diagram of the application, comprising both component, datatype, message, and activity definitions, (ii) define the components' behavior by means of state machines, (iii) relate the structural and behavioral parts by linking messages to events and activity pins, (iv) execute the model transformation that generates the C++ code skeletons of the activities and fill in the application specific code and compile it to a dynamic library, (v) execute the model transformation that generates the deployment model and modify it according to the application requirements, and (vi) run the application by executing the FraCC runtime with the deployment model and the dynamic libraries. Algorithms 6 and 7 show an excerpt of the FraCC implementation of the example shown in Figure 5.

From the perspective of the* MDSD expert*, transformations are as simple as in the iPOJO case, though they include more rules (twenty) and more lines of code (around three hundred and fifty).

We can conclude again that, in the same way as FraCC hides the details of the component implementation, the model transformation hides the details of FraCC.

## 5. Discussion

The aim of this section is to discuss about the benefits of the proposed approach. A way to demonstrate the usefulness of the approach is quantifying the benefits it provides in terms of time or cost investment needed to carry out the development of new applications or modifying the existing ones. However, cost estimation studies in the context of MDSD are rather scarce. There are many difficulties in performing cost estimation for MDSD-based approaches mainly due to the impact its use has on the software development effort itself and also due to the complexity of measuring varied artifacts that are generated and used throughout the process [48]. Nevertheless, there are many initiatives to estimate the cost of software development using COCOMO (COnstructive COst MOdel) models [49, 50]. This is the approach we follow in this section. COCOMO is an empirically based mathematical model that estimates the development effort according to the following expression:

(1) $$\begin{array}{c} E=\alpha \cdot S^{\beta }, \end{array}$$

where *E* is the development effort, *S* is the size of software artifacts involved in the development process, and *α* and *β* are empirical parameters that depend on the type of project and the selected COCOMO model. Specifically, *α* is in the interval [2.40, 3.60] and *β* is slightly greater than one, in the interval [1.05, 1.20]. As we consider the same development context in all the cases discussed below, the values of the parameters *α* and *β* do not change between the options.

In our proposal, we consider that *S* can be broken down into three terms.

- *S*_*a*_: the design of the application in terms of domain knowledge. For instance, the model in Figure 5.
- *S*_*b*_: the design of the application in terms of programming artifacts (classes, interfaces, functions, static and dynamic relationships, etc.). This is a software part that requires deep knowledge of programming paradigms and languages. For instance, the class diagram in Figure 6.
- *S*_*c*_: implementation of *S*_*b*_ by using available programming resources.

Note that there is a correspondence between C3 and *S*_*a*_, and between C1 + C2 and *S*_*b*_ + *S*_*c*_.

Thus, (1) now results in

(2) $$\begin{array}{c} E=\alpha \cdot {\left(S_{a}+S_{b}+S_{c}\right)}^{\beta }. \end{array}$$

If the development of such parts could be done completely independent of each other (i.e., as isolated projects), then the total effort could be reformulated as:

(3) $$\begin{array}{c} E^{\prime }=\alpha \cdot S_{a}^{\beta }+\alpha \cdot S_{b}^{\beta }+\alpha \cdot S_{c}^{\beta }. \end{array}$$

From (2) and (3), we can conclude that it is always better to develop and integrate several small artifacts than a big one (*E*′ ≪ *E*), since *S*_*a*_, *S*_*b*_, and *S*_*c*_ will be equal or lower in *E*′ than in *E*, given that the size and complexity of the developed application remain unchanged. Moreover, this partition allows that the projects can be carried out in parallel. In this sense, the approach is merely a formalization of the “divide and conquer” design principle. In fact, any approach that would allow developers to break down the global effort in independent projects will sure be an improvement. The problem is that this separation is not always easy to achieve because the software artifacts to be developed are often strongly coupled. Our approach enables to some extent this separation, as explained below.

In order to reason about the above equations we will consider two types of developer roles and the three development approaches considered in this paper. Regarding the roles, we consider “*application developers*,” which are experts in the application domain, and the “*transformation developers,*” which are experts in MDSD technology. The three considered development approaches are “*Traditional Development*,” “*MDSD development*,” and “*MDSD plus frameworks*.” For each option, we discuss next how *S*_*a*_, *S*_*b*_, and *S*_*c*_ could be addressed.

*Option 1: Traditional Approach.* The application developer (the only role present in this case) should address *S*_*a*_, *S*_*b*_, and *S*_*c*_, which in this case are hardly separable as described in Section 3.1. Developers should manage the domain concepts, model them and their relationships (as interfaces, classes, or functions), and implement the corresponding application. Thus, the effort estimation in this case is

(4) $$\begin{array}{c} E_{1}=\alpha \cdot {\left(S_{a1}+S_{b1}+S_{c1}\right)}^{\beta }. \end{array}$$

*Option 2: MDSD Development.* Application developers define the application in terms of CBSE concepts, which have a higher level of abstraction than in the previous case. The software design and implementation are performed by the expert in MDSD technology, which embeds them in a model transformation. This transformation takes as input the model defined before and generates executable artifacts. In this case, *S*_*a*_ can be done independently of *S*_*b*_ and *S*_*c*_. All the complexity of the implementation development is hidden to the application developer by the transformation. The effort estimation in this case is given by

(5) $$\begin{array}{c} E_{2}=\alpha \cdot S_{a2}^{\beta }+\alpha \cdot {\left(S_{b2}+S_{c2}\right)}^{\beta }. \end{array}$$

Now, *S*_*a*1_ and *S*_*a*2_ are similar in size, but in the second case *S*_*a*2_ is an independent project. The tasks performed by the MDSD expert (*S*_*b*2_ and *S*_*b*3_) are difficult to isolate. As shown in Figure 3, the design and implementation issues are strongly coupled in the transformations. The ultimate beneficiary from this option is the application developer, whose work and knowledge are limited to the CBSE domain which is expert. MDSD expert must address a complex work, which results in a model-to-code transformation that is rather difficult to maintain and reuse. This work can only be justified if the transformation is used to generate many applications. However, changes in nonfunctional requirements will require the modification of many rules of the transformation, which almost certainly will be coupled with others, producing a chain of modifications.

*Option 3: MDSD Approach Combined with Frameworks.* In this case, there are two possibilities: either there exists a framework that provides the nonfunctional requirements of the application, or an ad hoc framework should be developed. If such a suitable framework exists, the total effort is reduced to the following expression:

(6) $$\begin{array}{c} E_{3}=\alpha \cdot S_{a3}^{\beta }+\alpha \cdot {\left(S_{b3}+S_{c3}\right)}^{\beta }. \end{array}$$

The equation is formally identical to the previous case. The terms *S*_*a*2_ and *S*_*a*3_ can be considered equal, since from the point of view of the application developer both approaches provide the same support for application modeling. From the perspective of the expert in MDSD technology, the design and implementation of the transformation are again difficult to separate. However, with the new approach, *S*_*b*3_ + *S*_*c*3_ is smaller than *S*_*b*2_ + *S*_*c*2_, as the framework provides most of the implementation classes required to execute the application. It also offers a higher level of abstraction that hides the complexity of the underlying software artifacts (e.g., distribution management, concurrency, event management, etc.). As an example, the transformation described in Section 3 (which corresponds to the approach MDSD without frameworks) has about 2000 lines of code, while the transformations described in Section 4 (MDSD with frameworks) have about 200 lines of code in the case of iPOJO and 350 lines of code in the case of FraCC, that is, a ratio of around 1 to 7. The examples are from very specific experiences but we believe they are significant enough to show the benefits of the approach and the possibilities for developing more complex application with less effort.

In case that no framework supports the nonfunctional requirements and thus a new one should be developed, the effort expression is as follows:

(7) $$\begin{array}{c} E_{3}^{\prime }=E_{3}+\alpha \cdot S_{d3}^{\beta }. \end{array}$$

The new term *S*_*d*_ corresponds to the effort of developing an ad hoc framework. This effort can be seen separated from the rest of efforts and therefore appears as an additional term in the sum. This independence requires some explanation. The framework was not designed ignoring the MDSD technology. In fact, the design of FraCC was aimed at providing both an interpretation of the elements of the domain (C2) and “hot-spots” that facilitate the development of the transformations. But transformations were designed and implemented a posteriori, once the framework was done. Therefore, *S*_*d*_ can be performed independently of *S*_*b*_ and *S*_*c*_. The development of frameworks is not trivial and *S*_*d*_ can be very time consuming. Software engineers have to assess whether or not it is worth the effort. In our case, it was worth because FraCC is a relatively small framework and it had been designed to give long-term support to software development and research work in the domains of embedded applications and service robotics.

As it can be deduced, there are at least two more arguments in favor of the combined use of MDSD and frameworks. The first argument is that the probability of finding a framework that meets the requirements of an application is high. The most common scenario is not to develop a framework, but a software layer at the top of an existing framework or library that provides adequate C2 interpretation and appropriate “hot-spots.” In this case, the term *S*_*d*_ may be reasonably small. The second argument has to do with the development of frameworks and the interfaces they offer to their users. Frameworks are very powerful software artifacts but they are also often very large and complex. The MDSD technology can hide most of this complexity greatly reducing the learning curve of the frameworks.

For the shake of simplicity, we have only considered cases that target a single implementation framework for CBSE applications, but the approach can be generalized to integrate and reuse preexisting software artifacts. It is particularly representative of the case where we would like to integrate more than one existing framework, for instance, OSGi and Java Swing in order to develop complex graphical components. In this scenario, the framework developer would define all the modeling concepts needed to model such applications and create a layer that, on the one hand, instantiates the required frameworks and, on the other hand, provides the “hot-spots” the transformation will use.

Regarding software maintenance and evolution, the combination of MDSD and frameworks offers more advantages than their isolated use, since it is easier to cope with changes in nonfunctional requirements in the framework design than in model transformations. Many frameworks already include extensive support for configuring and tune the behavior of the applications. Also, there is a wide knowledge body on design patterns that can be used to include such mechanisms. Also, software updates (e.g., bug fix, addition of new features, etc.) have a lesser impact on clients that depend on frameworks than on clients that depend on model transformations.

## 6. Conclusions and Future Works

In this paper we have presented an approach that supports the development of component-based applications by using models and frameworks. It is based on the idea that frameworks can provide the run-time support and infrastructure required for executing component-based applications. Model-driven technologies provide the theoretical and technological support for modeling component-based applications from a high level of abstraction, as well as for generating the code that embeds both the application functionality and the selected framework instantiation.

The proposed approach has been illustrated by means of two representative case studies: (i) the integration of an already-existing, general-purpose framework, and (ii) the development and integration of an ad hoc framework for applications with real-time constraints. The former is the Apache iPOJO, an implementation of the OSGi R4 platform, which just requires the structural part of the components that make the application up, while the latter requires both component structure and behavior, since a complete control over the execution of the application is required in the target domain.

One substantial difference between this proposal and others analyzed in the section on the state of the art is the possibility of modeling applications using only the features and constructs offered by the CBSE approach. To achieve this, it is necessary to develop and reuse frameworks (implemented with OO technology) that provide the support for implementing these applications. MDSD is the approach that provides the conceptual and technological support both for the considered levels of abstraction and for the mechanisms for transformation from one to another. In short, the proposal described here offers the application developer a framework-based approach in which the primary artifacts of the development are component models, unlike other approaches that are programming language-oriented. Thus, the approach combines the benefits of modeling applications from a higher level of abstraction than objects, with the higher levels of code reuse provided by frameworks. We should highlight that the development of this proposal has been possible thanks to the technological maturity of frameworks and MDSD.

Finally, we have compared in terms of development cost this proposal with the canonical use of the MDSD approach in the literature, where a set of model transformations generate the whole implementation code. The comparison has been made from both qualitative and quantitative points of view, although estimation methods are not mature enough in the MDSD domain, and the proposal involves heterogeneous artifacts (models and OO frameworks). Despite the approach has been illustrated by means of a reduced set of case studies, the findings suggest that the following benefits can be obtained from its adoption: (i) ease of use of component-based frameworks since the user always develops a component-based model and does not have to learn the frameworks' internals; (ii) improved rates of application reuse since the same application can be implemented in different frameworks; (iii) separation of roles in the development team. Further research and integration of additional frameworks will allow us to consolidate these benefits.

Therefore, the lines of follow-up research currently in progress include the following: extending the available catalog of supported frameworks, incorporating cost models and metrics to further evaluate the proposal, and lastly conducting new case studies to demonstrate the integration of complementary frameworks such as graphic interfaces or communication middleware, to mention a few.

## Figures and Tables

**Algorithm 1:**
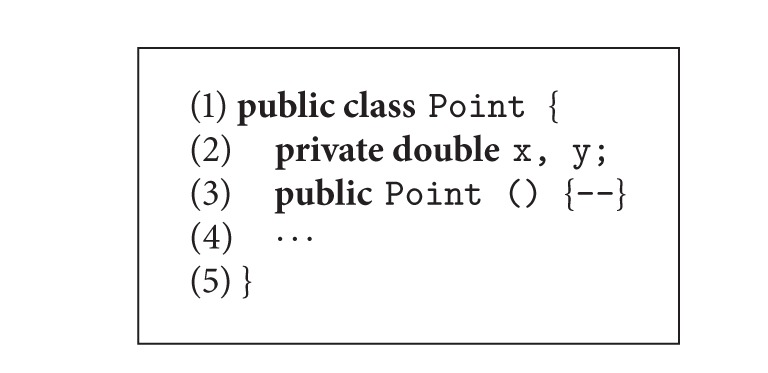
Skeleton of the OSGi implementation of the example shown in Figure 4 (implementation of the *Point* datatype).

**Algorithm 2:**
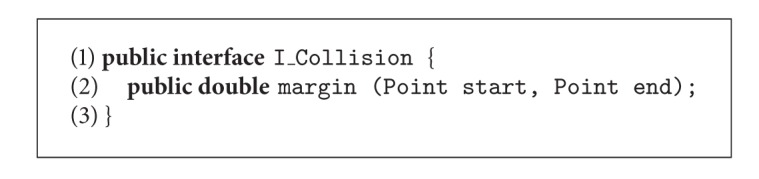
Skeleton of the OSGi implementation of the example shown in Figure 4 (implementation of the interface *I_Collision*).

**Algorithm 3:**
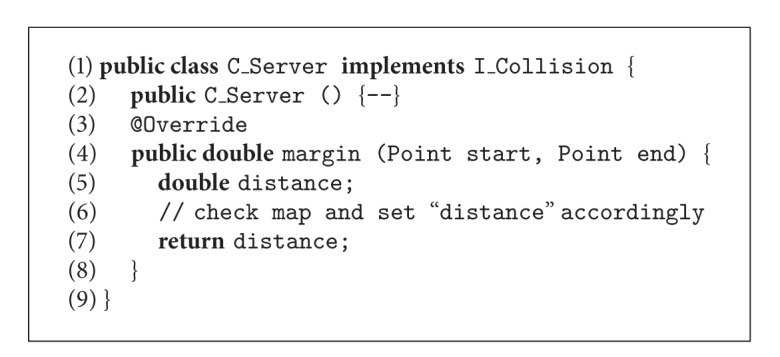
Skeleton of the OSGi implementation of the example shown in Figure 4 (implementation of component *C_Server*).

**Algorithm 4:**
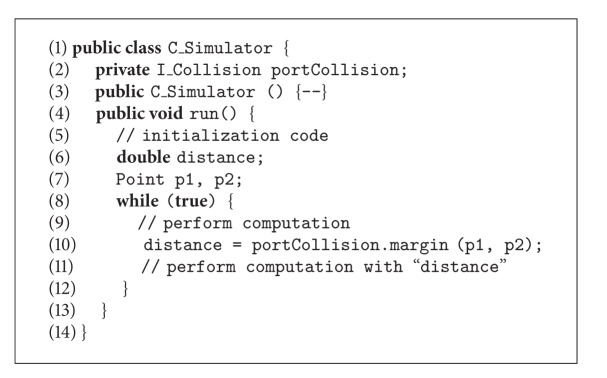
Skeleton of the OSGi implementation of the example shown in Figure 4 (implementation of component *C_Simulator*).

**Algorithm 5:**
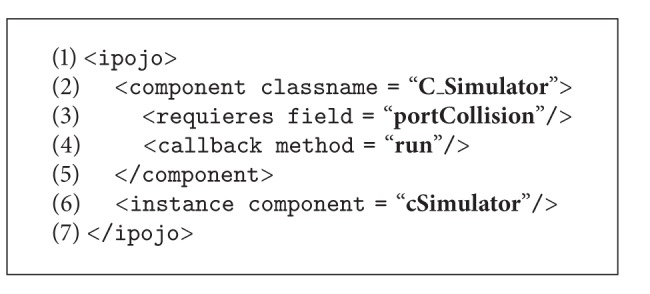
Skeleton of the OSGi implementation of the example shown in Figure 4 (description of the architecture).

**Algorithm 6:**
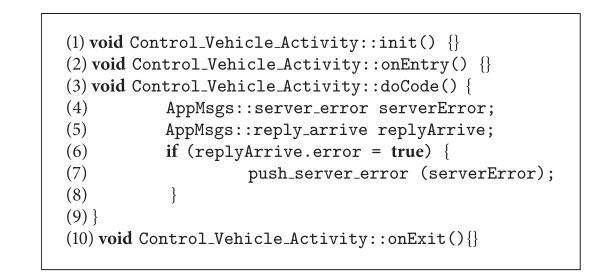
Skeleton of the FraCC implementation of the example shown in Figure 5 (excerpt of the implementation of the *Control_Vehicle_Activity* in FraCC).

**Algorithm 7:**
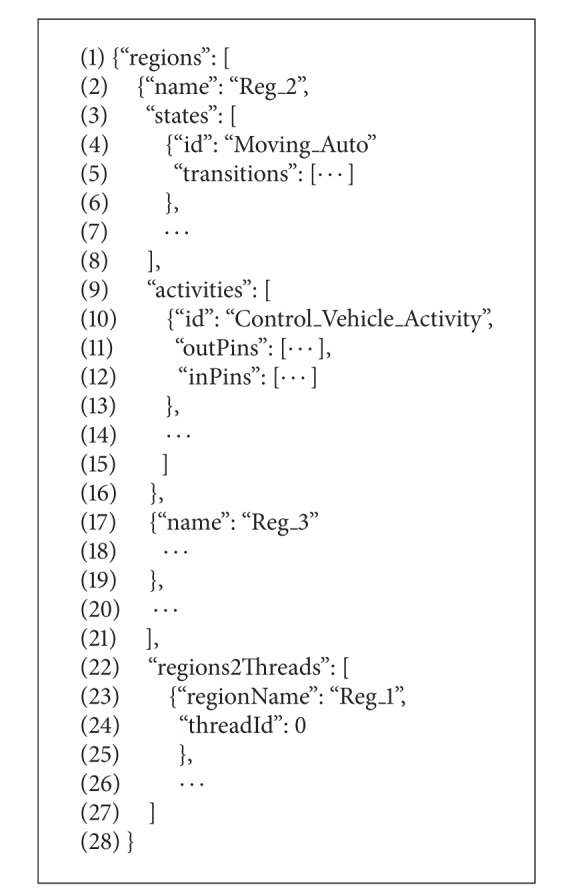
Skeleton of the FraCC implementation of the example shown in Figure 5 (excerpt of the JSON file describing the application architecture and its deployment).
